# A Pluralistic Perspective on Research in Psychotherapy: Harnessing Passion, Difference and Dialogue to Promote Justice and Relevance

**DOI:** 10.3389/fpsyg.2021.742676

**Published:** 2021-09-06

**Authors:** Kate Smith, John McLeod, Nicola Blunden, Mick Cooper, Lynne Gabriel, Christine Kupfer, Julia McLeod, Marie-Clare Murphie, Hanne Weie Oddli, Mhairi Thurston, Laura Anne Winter

**Affiliations:** ^1^School of Applied Sciences, Abertay University, Dundee, United Kingdom; ^2^Metanoia Institute, London, United Kingdom; ^3^Department of Psychology, Roehampton University, London, United Kingdom; ^4^School of Education, Language and Psychology, York St John University, York, United Kingdom; ^5^Department of Psychology, Faculty of Social Sciences, University of Oslo, Oslo, Norway; ^6^Manchester Institute of Education, Schools of Environment, Education, and Development, University of Manchester, Manchester, United Kingdom

**Keywords:** dialogue, epistemic (in)justice, pluralism, psychotherapy research, social relevance

## Abstract

The adoption of a pluralistic perspective on research design, processes of data collection and analysis and dissemination of findings, has the potential to enable psychotherapy research to make a more effective contribution to building a just society. A review of the key features of the concept of pluralism is followed by a historical analysis of the ways in which research in counselling, psychotherapy and related disciplines has moved in the direction of a pluralistic position around knowledge creation. Core principles of a pluralistic approach to research are identified and explored in the context of a critical case study of contemporary research into psychotherapy for depression, examples of pluralistically oriented research practices, and analysis of a pluralistic conceptualisation of the nature of evidence. Implications of a pluralistic perspective for research training and practice are discussed. Pluralistic inquiry that emphasises dialogue, collaboration, epistemic justice and the co-existence of multiple truths, creates opportunities for individuals, families and communities from a wide range of backgrounds to co-produce knowledge in ways that support their capacities for active citizenship and involvement in open democratic decision-making. To fulfil these possibilities, it is necessary for psychotherapy research to be oriented towards social goals that are sufficiently relevant to both researchers and co-participants to harness their passion and work together for a common good.

## Introduction

Psychotherapy research is conducted on a global scale, by investigators drawn from different occupations and disciplines, using a range of methodologies, and publishing in a large number of independent journals. As a result, notwithstanding a degree of influence exerted by governmental and other funding agencies, the psychotherapy research community can be viewed as comprising a largely self-organising open system that resists centralised direction. At the same time, it can also occasionally be valuable to evaluate the direction of travel of therapy research in terms of its contribution to broader societal objectives. In this paper, we suggest that although the field of therapy research has always reflected an implicit acknowledgement that the complexity of therapy outcomes and processes requires the adoption of a flexible, pluralistic approach to evidence, the full implications of a pluralistic perspective have yet to been fully articulated. More specifically, the predominant interpretation of pluralism that has been used by therapy researchers has not sufficiently taken account of crucial social and political aspects of this construct. By contrast, a more comprehensive application of a pluralistic stance in relation to therapy research has the potential to significantly enhance the contribution of psychotherapy, and related practices, to addressing contemporary social issues.

Pluralism is a philosophical and ethical tradition based on the idea that there is no single perspective or truth that is universally valid (Rescher, 1993). Pluralism represents an acknowledgement of the ultimate impossibility of reducing the interconnectedness, complexity and uniqueness of life to a set of laws or theories. From a pluralistic stance, human experience and forms of life, across all cultural traditions, can be seen to have been characterised by dissensus rather than consensus. Awareness of the existence of a diversity of perspectives, along with a never-ending effort to reconcile such differences, has been a fundamental aspect of both individual and societal development in modern societies (Taylor, 1992). Important aspects of a pluralistic stance include a commitment to dialogue as a means of bridging different perspectives and to a cognitive style that emphasises ‘both/and’ or ‘and/and’ rather than ‘either/or’ dichotomous thinking.

The authors of this paper are aligned with a collaborative framework for therapy practice, known as *pluralistic therapy* that represents a systematic attempt to develop a form of practice informed by a pluralist philosophy (Cooper and McLeod, 2011; Cooper and Dryden, 2016; McLeod, 2018; Smith and de la Prida, 2021). A key principle of pluralistic therapy is that different people are likely to be helped by different things at different times. Problems in living for which individuals and families seek therapeutic help are viewed as arising from complex interactions between multiple life events and sources of adversity. Pluralistic practice addresses these problems by making use of strategies and methods from multiple sources, including supportive and healing practices available within the community. The process of client-therapist collaboration is organised around clarifying what the client wants to use therapy to achieve (their goals), identifying specific tasks that might contribute to step-by-step progress in the direction of goals and agreeing methods for accomplishing these tasks. The therapist functions as a facilitator or orchestrator of dialogue and shared decision-making around finding and assembling ideas, activities and ways of communicating and relating, suggested by either the client(s) or therapist(s), or emerging in the work itself. Procedures for supporting client-therapist collaboration and shared understanding include therapist transparency around what they can offer, techniques for elicitation of client preferences (Norcross and Cooper, 2021), and active elicitation and application of the client’s knowledge through experience and cultural resources. Strategies for ensuring that therapy remains in alignment with client goals include routine use of metacommunication, process and outcome feedback tools and design tools, such as collaborative case formulation mapping. Pluralistic therapy does not comprise a fixed or static theory or set of procedures. Rather, it comprises an open system and community of practice that encourages dialogue, innovation and sharing of experience.

Over the past decade, the priority of the pluralistic therapy community has been to establish structures for training, practice and supervision to support members in working collaboratively with clients. An important strand of that effort has been the development of a research base that would identify evidence to facilitate the development of pluralistic practice; conducting studies on the process and outcomes of pluralistic therapy itself (e.g. Cooper et al., 2015; Di Malta et al., 2020). The intention has been to build an approach to therapy that is research-informed rather than research-directed (Hanley and Winter, 2016), based on the principle that effective practice draws on multiple ways of knowing: ethical, personal, theoretical, cultural and scientific (McLeod, 2016).

In the course of developing an evidence base for pluralistic therapy, we gradually came to realise that we were beginning to see the psychotherapy research literature as a whole from a distinct pluralistic perspective. In particular, we came to believe that the more pluralistic a research study or programme was, the more useful it was for practice, and the more likely it was to make a contribution to social justice.

The present paper builds on earlier work by Hanley and Winter (2016), in seeking to explore and further refine the nature of a pluralistic perspective on psychotherapy research. A historical overview is provided around how the concept of pluralism has been used in psychotherapy research. This is followed by a summary of key principles of a pluralistic perspective on research, and then, a case example that examines how a more explicitly pluralistic approach might enhance the relevance of research in relation to an area of inquiry that has comprised a central focus of psychotherapy research in recent years. The paper concludes by looking at possible ways in which a pluralistic perspective might be realised. Our aim, in all of these areas, has been to consider the implications of a pluralistic perspective in relation to the field of psychotherapy research in general, rather than solely in respect of pluralistic psychotherapy as a specific therapy orientation.

## A Historical Perspective on the Influence of Pluralism in Research in Counselling and Psychotherapy and Related Disciplines

From the start, psychotherapy research has been primarily based in the ideas and methods of psychology and psychiatry – disciplines that have historically prioritised quantitative and experimental approaches to research. Nevertheless, despite these disciplinary pressures, the first generation of therapy researchers, notably Carl Rogers and Hans Strupp, sought to create a flexible and responsive methodology for the study of psychotherapy that was consistent with its existence as a complex, co-constructed, agential and interpersonal form of practice. For example, the research group led by Rogers made use of case study methods, qualitative methods and projective techniques (Rogers and Dymond, 1954). Strupp and Hadley (1977), arguing that it was essential to understand therapy outcomes from multiple perspectives (client, therapist and society). However, from the 1970s, the increasing societal profile of psychotherapy, and in particular its growing presence with state-funded healthcare systems, meant that major sectors of practice came to be controlled by assumptions, policies and procedures associated with neoliberal political and economic ideology, and the implementation of these ideas through the adoption in public sector organisations of management philosophies that emphasise competition rather than collaboration (McLeod, 2016; Sundet, 2021). For the psychotherapy research community, this shift was reflected in the reification of schools of therapy as products in a crowded mental health marketplace, and the adoption of randomised clinical trial (RCT) methodology as a means of determining ‘winners’ and ‘losers’ within a competitive environment. Within this new approach, studies of psychotherapy effectiveness increasingly adopted a single primary outcome measure, typically in the form of a client self-report symptom scale.

From the 1980s, the concept of pluralism began being used in the psychotherapy literature to signal resistance to the hegemony of RCTs and empirically validated and manualised therapies (Omer and Strenger, 1992; Samuels, 1995; Downing, 2004). In the context of research, the idea of *methodological pluralism* was introduced in an influential paper by Howard (1983), as a means of legitimising the use of a wide range of methodologies alongside clinical trials. While Howard was commenting specifically on the value of case study methods, later contributions extended the argument to include qualitative methods (Mearns and McLeod, 1984; Goss and Mearns, 1997; Slife and Gantt, 1999; Barker and Pistrang, 2005). While acknowledging that different methodological approaches (quantitative, qualitative and case study) were grounded in contrasting epistemological positions and values and associated with different quality criteria, these writers argued that different research questions were most effectively addressed by different methods and that the study of psychotherapy required the use of multiple research approaches. Important themes within the argument for methodological pluralism were that convergence of findings across methodologies had the potential to reinforce the credibility of research and that evidence hierarchies that gave higher weighting to meta-analyses of findings from RCTs were misguided. Over the years, the principle of methodological pluralism, understood as tacit acceptance of the value of different methodologies, has become widely accepted within the psychotherapy research community.

The vision of methodological pluralism advocated by Howard (1983) and others was primarily formulated as a set of broad principles, with limited practical guidance on how to handle different types of data in the process of conducting an actual study. These technical issues troubled researchers, particularly those whose initial training had focused on quantitative designs and techniques. As a consequence, there began to emerge a literature around the use of *mixed-methods research* (MMR) designs (Haverkamp et al., 2005). An underlying driver within the MMR literature has been a wish to combine the strengths of both qualitative and quantitative methods. As a consequence, authors have highlight concerns and challenges associated with combining multiple methods in one study and emphasised the necessity for having a clear understanding of the distinctive contribution of each approach around such domains as: quality standards for different styles of data collection and analysis; underlying values and epistemological assumptions; and reporting formats. There are many different methodologies that can be combined in different ways for different purposes which have stimulated a proliferation of MMR texts and models (Tashakkori and Teddlie, 2010; Creswell and Plano Clark, 2011). Recent years have seen a steady growth in interest in MMR research on psychotherapy topics, including formulation and dissemination of APA guidelines (Levitt et al., 2018).

As MMR research became more established, it became increasingly apparent that many MMR studies operate not primarily on the basis of a pre-determined research design that is then followed through, but on a collaborative process between a team of researchers each of whom represents a distinctive methodological competency, conceptual/theoretical perspective or area of lived experience (a useful discussion of this theme can be found in Wachsmann et al., 2019). A key element of this aspect of the real-life implementation of MMR research is that taking methodological pluralism seriously requires making use of people who are immersed in each approach or represent different perspectives, rather than depending on the less intense understanding of contrasting approaches and perspectives that might be available to a single research generalist. An additional area of methodological learning within the recent critical and reflective literature on MMR has been that successful studies pay particular emphasis on areas of difference (across methodologies, participants and theories), as well as areas of convergence, within the process of data collection and analysis (Johnson, 2017).

The next step in the historical evolution of a pluralistic approach in research in psychotherapy and related disciplines has therefore comprised the development of attention to dialogical processes, described by Johnson (2017) and colleagues as a form of *dialectical pluralism* that as:

…asks all of us to appropriately listen to what needs to be listened to for each research question, purpose, stakeholder interest, and practical activity. This broad dialecticalism will enable people to continually interact with different ontologies, epistemologies, ethical principles/systems, disciplines, methodologies, and methods in order to produce useful wholes. The process should continually build on what we know and feel and value now and produce new, dialectically derived, “knowledge(s),” programs, theories, and deliberative democratic human coalitions. (Johnson, 2017, p. 158)

Johnson and Schoonenboom (2016) present an account of what dialectical pluralism looks like in practice, through exemplary studies on various aspects of healthcare practice. A common pattern of these studies is that researcher beliefs and knowledge (e.g. what they have found in an initial study, such as an RCT) are rigorously exposed to critique by relevant stakeholders (e.g. patients and healthcare providers), leading to a new shared understanding that can then form the basis for further cycles of inquiry. While dialectical pluralism is similar in some respects to a broader acknowledgement of stakeholder perspectives in healthcare research and realist evaluation (O’Cathain et al., 2019), it goes further in calling for a systematic and disciplined capacity for listening, reflexivity and openness to difference on the part of researchers. Detailed accounts have been developed of techniques and strategies used by dialectical realist research teams to support the adoption of a dialectical or dialogical practices (Johnson and Schoonenboom, 2016; Johnson, 2017).

A complementary research tradition that similarly incorporates a pluralistic and dialogical ethos can be found in studies that have adopted participative action research and collaborative inquiry approaches (Ponterotto, 2005; McLeod, 2001). Such studies reflect a position that the primary aim of research should be to facilitate change in terms of promoting equality and social justice, empowering individuals and communities and solving real-world problems. For these researchers, the capacity to achieve such outcomes is the real test of the value or validity of a research study. This approach to research can be understood as a form of ‘engaged’ inquiry (Chevalier and Buckles, 2019), influenced by the ideas and values promoted by key 20th century thinkers, such as Paulo Freire, Jurgen Habermas and Kurt Lewin (Reason and Bradbury, 2013). It is also consistent with ideas of ‘collaboration as a matter of principle’ outlined as part of the psychotherapy social justice agenda (Winter, 2019, p.180). Typically, service users or community members may be involved in the design of a study, collection and analysis of data, writing and dissemination, for example in the programme of collaborative research that has involved service users and therapists working together to establish a basis for more effective therapy and recovery interventions in bipolar disorder (Veseth et al., 2012; Billsborough et al., 2014). A similar approach to a collaborative, emancipatory approach to inquiry can be found within the methodological tradition associated with critical psychology (Teo, 2015; Fine et al., 2021; Levitt et al., 2021a,b).

Co-production is a further example of a pluralistically oriented form of research that promotes engagement and shared decision-making between researchers and participants. Drawing from the analysis by Ostrom (1990) of the operation of systems of common ownership (Ostrom, 1990), co-production is a justice-based approach (Cahn, 2000) that has been widely applied within national health and social care services in the United Kingdom to empower citizens to become participatory agents in their own care (Coote, 2002; Needham and Carr, 2009; Worsley et al., 2021). Commitment to co-production is now a central funding criterion of the UK National Institute of Health Research, on the grounds that all research should be carried out ‘with and by patients, rather than to or for them’ (NIHR, 2021a,b).

As with the development of co-produced interventions in healthcare, co-produced research ideally involves the equal and reciprocal co-creative involvement from design, through action and into dissemination, with research partners drawn from a range of backgrounds and roles. The inclusion of multiple vested stakeholders in the design and implementation of research, allows for greater complexity of understanding of both the phenomenon at hand, and the nested systems within which it operates (Gibert et al., 2010; Conte and Davidson, 2020). Additionally, the involvement of service users to develop relevant and timely research questions may help to reduce the widespread research-practice gap noticed especially in mental health research.

Co-productive research is driven by a commitment to a pluralistic stance that emphasises inclusion of multiple stakeholders and perspectives and mutual trust between research partners. The process of engaging in co-produced research has been termed as ‘turbulent’ and ‘challenging’ (Worsley et al., 2021) due to the complex interpersonal dynamics that can arise when professionals and patients are asked to work together in partnership. Co-production research partners must commit seriously to *hermeneutic justice* (Fricker, 2007), in which ways of understanding conveyed by different actors are considered to have equal value, especially where the voices of some actors have been previously silenced (Blunden and Calder, 2020). Examples of co-produced psychotherapy research studies include Blunden (2020) and Curran et al. (2021).

These issues have inevitably led pluralistically oriented therapy researchers to look towards theory, research and practice around decolonisation as a source of understanding around how to handle such issues. A decolonial perspective involves facing up to deeply entrenched areas of injustice in contemporary society that are rooted in large-scale, violent historic exploitation of indigenous peoples and the lands on which they lived. Supported by the work of researchers, scholars, activists and artists in a wide range of disciplines in all parts of the world, this broad approach involves interrogating the roots of injustice and oppression, challenging existing power relations, achieving restorative justice and building postcolonial discourses and communities (Goodman and Gorski, 2015; Barnes, 2018; Smith, 2021). One of the consequences of colonialism has been not only the unequal distribution of material goods and power, but also the fact that the majority of those in power does not recognise themselves as such but is still playing their part in larger discourses and abstract systems, such as patriarchy, privilege or in what is termed as ‘normal’ (e.g. neurotypicality and heteronormativity). Current movements and frameworks, such as Black Lives Matter, #MeToo, or critical psychology and intersectionality, are about finding one’s voice and amplifying the voices of marginalised others. Pluralistic inquiry can engage with this by asking questions inspired by postcolonial theory that address alienation, power differences and silenced voices. In recent years, even though some researchers and practitioners have begun to develop a postcolonial approach to psychotherapy research, it is clear that more needs to be done. For example, although trauma therapy has received considerable attention from a postcolonial perspective (Bennett and Kennedy, 2003; Andermahr, 2016), traumatic experiences of minorities are still marginalised while Western definitions of trauma are taken as universally valid (Craps, 2013).

This historical analysis has sought to provide an outline of how methodologies that reflect an explicit or implicit pluralistic standpoint have emerged over several decades as increasingly salient aspects of research in psychotherapy, counselling and related disciplines. The earliest references to methodological pluralism represented a response to the methodological hegemony of measurement and experimentation in research. Pluralism was put forward as a solution to the perceived limitations of relying solely on quantitative research approaches. These sources used the concept of plurality as a synonym for multiplicity and diversity, often within the conclusion section of an article or chapter, as something to be accepted and move towards (see, for example Rieken and Gelo, 2015). Over time, a pluralistic perspective began to move on from arguments about the legitimacy of qualitative and case study approaches to technical solutions to the challenge of combining different kinds of data. The most recent phase has been marked by the establishment of a distinctively dialogical, collaborative and co-production approach to the creation of practical knowledge in psychotherapy and allied disciplines, and then most recently to common purpose with political and scholarly initiatives around decolonisation. The underlying dynamic in this process has been a shift from interpreting pluralism as a form of respectful relativism that acknowledges the co-existence of different points of view to a more active stance that attends closely to difference as a source of learning and insight. This transition has required researchers to engage with uncomfortable and often emotionally troubling differences associated with power, colonialism, unearned privilege and other inequality fault-lines in contemporary society.

## Principles of a Pluralistic Perspective on Psychotherapy Research

Although the development of a pluralistic perspective on research in psychotherapy has been based in the work of individuals and groups influenced by different conceptual frameworks and operating in different contexts, it is possible to identify some shared underlying methodological assumptions and practice implications.

### Methodological and Epistemological Flexibility and Inclusiveness

A key principle of a pluralistic perspective on research is an appreciation that all ways of knowing and sources of knowledge have something to offer. Pluralistic inquiry does not define itself in opposition to other research traditions or consider any such traditions to be ill-founded. Instead, all forms of inquiry are regarded as possessing their own distinctive strengths and limitations. Pluralistically oriented psychotherapy research does not promote qualitative research over RCTs or neuropsychological studies, favour wholism and emergence over reductionism or vice-versa. An important study in relation to this topic was conducted by Levitt et al. (2020) who interviewed leading psychology researchers from a wide range of methodological traditions, around their stance in relation to the adopting a detached, objective research attitude or espousing the use of disciplined subjectivity. A striking finding from these interviews was that all of the research participants regarded both objectivity and subjectivity as serving valuable scientific purposes and had made use of their personal capacities for subjectivity and objectivity as necessary over the course of their careers.

A pluralistic perspective on research seeks to operate from the kind of both/and stance represented by informants in the Levitt et al. (2020) study. This principle represents a central implication of the ethical implications of espousing a pluralist view of reality: if different individuals and groups hold contrasting beliefs about what is true, the ethical choice is between discounting, ignoring or suppressing the beliefs of others or engaging in dialogue that seeks to make bridges between alternative ways of thinking. All of the pluralistically inclined research traditions discussed earlier in this paper reflect the latter ethical choice and can be regarded as invitations to move beyond established positions in ways that have the potential to broaden and fuse horizons.

Influential figures in the psychotherapy research community have argued that contemporary psychotherapy research and practice are dominated by a stultifying theoretical and methodological ‘monoculture’ (Leichsenring et al., 2018, 2019) and that a pluralistic perspective should be regarded as existing as a focus of opposition to such hegemonic tendencies. This is not the inclusive and invitational position adopted in the present paper, which views the psychotherapy research community as comprising many vibrant ‘micro-cultures’ that would benefit from talking to each other a bit more, in ways that would allow us all to learn with and from each other. The fact that large psychotherapy providers, such as government health departments and managed care organisations, might seek to impose uniformity around therapy services that are offered to the public is an indication that psychotherapy research might benefit from adopting a more pluralistic approach that takes political, social and historical and social factors into account.

### Expect – and Welcome – Multiple Credible Answers to the Same Question

From a pluralistic perspective, research analyses and conclusions that yield multiple answers (divergence/dissensus) are of equal value to those that generate convergence/consensus. Pluralistically oriented research reports and reviews highlight different interpretations of data (e.g. by an auditor or co-researcher in a qualitative study, through application of alternative statistical techniques and attention to outlier cases) as having potentially significant implications for understanding, research, practice and theory development. Diverging perspectives or findings arising from different data sources or participants are viewed as steps in a dialectical process that has the potential to lead to a new (or more differentiated) theory or synthesis (Levitt et al., 2020). The existence of multiple ‘truths’ is not only a core philosophical assumption of pluralism but also is a routine aspect of the practice of psychotherapy: much of the process of therapy is based on the creation of meaning bridges, empathy and ways of talking and connecting that have the effect of allowing people to function within a multi-voiced intra- and interpersonal reality. By corresponding more closely with everyday experience, multiple answers to a research question have the potential to make findings not less, but more relevant for policy and practice.

### Active Promotion of Epistemic Justice

Within both the natural and social sciences, there are multiple ontological and epistemological positions that are utilised in the service of legitimate inquiry. There also exist highly significant knowledge structures within society as a whole, for example in respect of spiritual and faith beliefs, and indigenous systems of knowledge, that operate independently of scientific empirical knowing. In everyday life situations, participants make use of multiple ways of knowing alongside scientific evidence, for example personal experience, knowledge arising from membership of a culture or occupational group, ethical values, theoretical understanding and narrative knowing. Psychotherapists and clients routinely operate within and across these alternative ways of knowing. In relation to psychotherapy research, these factors mean that it makes little sense to regard any single source of knowledge (e.g. RCTs or meta-analysis of RCTs) as offering a reliable guide to practice or decision-making. Instead, practical decisions should be based on a balanced and informed appraisal of all available sources of evidence.

Occasions when someone in authority (e.g. a therapist or policy-maker) unilaterally prioritises one source of evidence over another should be viewed as episodes of epistemic injustice and misuse of epistemic privilege. Fricker (2007) identified two forms of epistemic injustice: *testimonial injustice*, where evidence provided by a person is not taken seriously because of who they are (e.g. a client’s evaluation of therapy being disregarded because of their alleged diminished capacity for rationality) and *hermeneutical injustice* when a source of evidence is not well enough understood at an institutional or organisational level for it to be taken into account (e.g. when journal reviewers reject qualitative research manuscripts because of lack of knowledge of qualitative methodology). Epistemic injustice has been identified as highly prevalent in mental health settings, for instance in terms of lack of credence given to the cultural and experiential knowledge of service users, and black and minority ethnic staff and clients (Carel and Kidd, 2014; Crichton et al., 2017; Kidd and Carel, 2017; Newbigging and Ridley, 2018). Epistemic injustice may also occur with research groups, for example when qualitative data are analysed by members of a research team that includes novice researchers alongside senior academics, or individuals from different cultural or social class backgrounds (Levitt et al., 2021a).

A pluralistic perspective on research pays particular attention to strategies for prevention of epistemic injustice through relevant design, data collection and analysis, and dissemination, and intentional choice of research topics and questions intended to address previous epistemic injustice (e.g. carrying out research in collaboration with members of marginalised groups).

### Dialogue as a Criterion for the Validity, Credibility, Trustworthiness and Practical Utility of Research Conclusions

Scientific research is an essentially collective process, that depends not only on the capacity for imagination and rational thinking of individual researchers, but on the capacity of a set of findings to enter and survive the process of dialogue with other, independent members of a scientific community, in the form of critical responses or readers and reviewers, replication studies and data re-analyses (Brown, 2012; Stuckey et al., 2015). Because therapy research is fragmented into sub-communities (e.g. groups who study psychodynamic therapies, or CBT, or humanistic therapies), most research reports are only read by those who are already broadly predisposed to agree with what is being reported (McLeod, 2017). In addition, major groups of possible stakeholders who might have a view on the findings of a study, such as practitioners and clients, rarely or never read research papers. In some qualitative research papers, even though data and findings may be made available for comment by independent research auditors, or research participants, the ensuing dialogue with researchers is seldom reported. Taken as a whole, these scenarios mean that therapy research studies are scrutinised to a very limited extent. By contrast, the practice of both pluralistic therapy and pluralistic research relies on a process of putting difference to work through treating contrasting perspectives as opportunities for learning (Johnson, 2017). Both pre- and post-publication open dialogue around research reports have the potential to produce findings that are more nuanced and relevant to practice (Nosek and Bar-Anan, 2012). While the broader scientific community has found it hard to sustain such initiatives (Wakeling et al., 2019), there are sufficient motivated and interested practitioner and service user readers to make such an approach feasible.

### Doing Research That Is Oriented Towards the Accomplishment of Social Justice Goals

Pluralism is associated with a pragmatic philosophical stance in its emphasis on evaluating the success of any actions in terms of criteria that are decided at a local level, rather than on the basis of abstract or universal criteria (Fishman, 1999; Hanley and Winter, 2016). In pluralistic therapy, for example the process of therapy and the final decision on whether it has been helpful are anchored in goals identified by the client. Similarly, one of the implications of a pluralistic perspective on research is that an important criterion for evaluating studies should be in terms of the difference that they make in relation to social needs and goals that are meaningful to individuals and communities. An example of the difference between research that is personally and theoretically meaningful, as against aiming to address social injustice, can be found in a programme of research into the role of counselling and other forms of emotional support for people experiencing sight loss. This programme originated in a stand-alone grounded theory qualitative study of the emotional impact of sight loss (Thurston, 2010). The experience of conducting this study, and in particular the response of others to its publication, opened a specific societal goal (development of emotional support services for sight loss) that served to guide the direction of further work. Further studies drew on other methodologies, such as case study analysis (Thurston et al., 2013) and surveys (Thurston and Thurston, 2013; Pybis et al., 2016). Because the social significance of this research was apparent to individuals with sight loss, health professionals, third sector vision impairment organisations, researchers from other disciplines and politicians, it became able to draw on an expanding network of collaborative consultation and dialogue, and to co-produce training courses for counsellors and other helpers. Many other similar examples of research programmes oriented towards social justice goals could be identified.

Authentically pluralistic and inclusive research that involves collaboration, co-production and dialogue is more likely to occur in situations in which a programme of research is organised around a social goal that is sufficiently broad and practically significant, and whose relevance is sufficiently widely acknowledged to energise the passion, active involvement and passion of individuals and groups beyond the academic community. Such situations enable research partners to bring their own sources of power into a project. In such research contexts, the concept of passion refers to the capacity of participants to be motivated by a goal that transcends their own individual interests, draws on all aspects of who they are as a person and calls for sacrifice in the service of a greater good (Duffy et al., 2013).

The methodological principles outlined above, derived from philosophical and social usage of the concept of pluralism, as well as the range of pluralistically oriented research traditions already discussed, provide a preliminary guide or checklist for thinking about how to incorporate a pluralistic perspective into research in psychotherapy.

## Pluralistically Oriented Therapy Research: Illustrative Case Examples

Within the field of psychotherapy research, although there are few studies that have explicitly espoused a pluralistic perspective, it is possible to use pluralistic principles to develop an understanding of what might be missing in the ways that studies and reviews are conducted. In the following sections, the area of research on psychotherapy for depression is used to explore some of the ways in which a pluralistic perspective makes it possible to begin to see how dialogical and collaborative approaches might enhance the practical utility of personal and institutional investment in psychotherapy research into this major mental health issue. The focus then turns, more briefly, to consideration of the relevance of a pluralistic perspective to methodological challenges around collaboration in research, investigating culturally-responsive therapy and conducting pluralistic systematic reviews.

### Pluralizing Depression

Apart from its inherent significance as a major area of therapy theory, research and practice, the topic of psychotherapy for depression is of interest from a pluralistic perspective because it has been the recent focus of critical scrutiny in the United Kingdom that makes it possible to identify different ways of thinking about evidence, and significant failures in dialogue. In the United Kingdom, the National Institute for Health and Care Excellence (NICE)^1^ is an independent, government-funded organisation that publishes clinical guidelines to support clinical decision-making in physicians and other practitioners in relation to a wide range of health conditions and aspects of social care. Guidelines are informed by an evidence hierarchy in which systematic reviews of RCTs are given the highest weighting and are updated on a regular basis. NICE guidelines are regarded by clinicians in many countries as demonstrating exemplary standards of rigour. In 2009, NICE published a set of guidelines on the treatment of depression, which recommended a range of psychological therapies that might be used for different degrees of symptom severity. In 2017, following an extensive period of consultation and a systematic meta-analysis of new evidence, it produced a draft of an updated depression guideline, which was circulated to stakeholder groups for comment. Despite the fact that the review process and guideline recommendations were formulated by leading figures within the research community, the 2017 draft revised guidelines (which strongly favoured CBT) were widely rejected by key stakeholder groups, including psychologists, psychiatrists, general practitioners, counsellors, psychotherapists and service user organisations (see, for example Barkham et al., 2017; McPherson et al., 2018; Thornton, 2018). The central issue, for critics, was that the procedure had not been sufficiently pluralistic. In particular, application of an evidence hierarchy that placed the highest value on RCTs and systematic reviews of RCT findings was regarded as having had the effect of omitting or downgrading crucial sources of evidence, such as qualitative client experience studies and naturalistic studies of routine therapy practice in everyday therapy settings.

The critical response to the draft NICE depression guidelines is consistent with findings from a study of a different NICE guideline project, that, in itself, RCT evidence is not necessarily regarded as reliable and trustworthy, even within groups of senior researchers who have spent their careers conducting such studies (Brown et al., 2016). The issue of trustworthiness was further explored in relation to RCT evidence relating to psychotherapy for depression in a unique investigation conducted by McPherson et al. (2020). In this study, groups of people with an interest in mental issues (mainly service users, carers and GPs) received a detailed presentation on a psychotherapy for depression RCT study and then invited to share their reactions to what they had heard. None of the participants were convinced that the study that had been described to them helped them to understand therapy for depression or provided information that might help them to decide whether or not such therapy might be relevant to them or to other people they knew. They regarded the RCT design as over-simplifying a complex set of issues and generating ‘headline messages’ that were misleading. Participants in the McPherson et al. (2020) study also had many suggestions about the type of study that might be more relevant. From a pluralistic perspective, the significance of this study lies in its demonstration that lay people with personal experience of mental health issues are capable of contributing to dialogue around the pros and cons of different types of research, if provided with an appropriate setting within which such conversations can take place.

Another area in which a pluralistic perspective highlights problematic aspects of research on therapy for depression is concerned with how depression is defined, measured and understood. Depression outcome studies typically evaluate the effectiveness of treatment using self-report symptom measures completed by clients, such as the Beck Depression Inventory (BDI) or Patient Health Questionnaire (PHQ-9), at the beginning and end of therapy. Historical analysis by McPherson and Armstrong (2021) has demonstrated that the concept of depression embodied in these measures has become narrower over time. Other studies have analysed qualitative evidence around how clients, their family members and therapists decide whether therapy has had an effect on depression and has found that these stakeholders make use of a much wider set of outcome criteria than those deployed in research studies (Catchpole, 2020; Chevance et al., 2020; De Smet et al., 2020; Krause et al., 2020a,b). Research into the experience of depression in everyday life has found that lay people possess complex and highly differentiated discourses and frameworks for making sense of recovery from depression (Hänninen and Valkonen, 2019; Llewellyn-Beardsley et al., 2019; Bear et al., 2021), including a range of possible pathways to change (Valkonen et al., 2011). Finally, studies in non-Western cultures observe important differences between the ways that depression is understood in these settings, and the measures used by therapy researchers (Haroz et al., 2017; Vink et al., 2020). Looking at the ways in which depression is measured and understood in therapy research as a whole, it seems apparent that researchers are missing potentially important aspects of the phenomenon they are investigating, and not taking sufficient account of differences between professional and everyday ways of understanding depression. By contrast, a pluralistic orientation to research would suggest that these are crucial areas of investigation for producing a practically relevant evidence base around how to help people to move on from depression. Stänicke and McLeod (2021) provide an example of how attention to these forms of difference and paradox may be used to stimulate new directions in therapy research.

Research into therapy for depression predominantly reflects a narrow focus on the process and outcomes associated with specific depression-related aspects of what happens in the therapy room. However, therapy for depression rarely occurs in isolation. Most clients who are depressed report other co-existing problems and issues (Morrison et al., 2003). Clients make use of other forms of help alongside seeing a therapist (Wilson and Giddings, 2010). Family members are involved in a myriad of ways, whether or not the therapist every meets them face to face (McPherson and Oute, 2021). A large proportion of clients has made use of antidepressant medication in the past or is on medication while receiving therapy. These activities are likely to have exposed them to a ‘chemical imbalance’ explanation of depression that may be difficult to reconcile with therapy (France et al., 2007; Kemp et al., 2014). The sequencing of therapy and medication may follow different pathways. Some clients turn to therapy when medication has not helped, and they have reached rock bottom (Wells et al., 2020). Others regard medication as energising them sufficiently to engage with psychotherapy (Cartwright et al., 2018). A pluralistic perspective highlights the significance of these (and many other) aspects of therapy for depression that transcend a specific therapy focus or depression focus.

The kind of critical social analysis that is entailed by a pluralistic perspective invites analysis of how differences in power and status have shaped contemporary approaches to research into psychotherapy for depression. The emergence of depression as a major mental health issue, in the 1950s, arose from the efforts of drug companies to develop markets for new products (Healy, 1999, 2006; Greenberg, 2010). These initiatives involved incentives to family physicians to diagnose patients as depressed, direct marketing to members of the public and funding for psychiatrists to revise the diagnostic manual of the American Psychiatric Association to highlight a medicalised concept of depression (Davies, 2021). A Western medicalised understanding of depression was exported to other countries worldwide. For example, Kirmayer (2002) described the intensive drive to promote antidepressants in Japan, in the face of considerable local opposition. As psychiatric diagnoses became established as the primary organising principles for mental health provision and conditions for reimbursement and employment, counsellors, psychotherapists and psychologists gradually integrated medical terms, such as depression, into their research and practice. Because diagnosis operates on a universalistic basis in which everyone’s problems are categorised in the same way, it became harder to talk about differences arising from culture, social class and gender. Although psychotherapy for depression does not share the brutal history of colonial exploitation of non-European peoples, the pluralisation of this area of practice has much to learn from decolonising approaches to research (Kiddle et al., 2020; Smith, 2021).

This case study of research into psychotherapy for depression illustrated the limitations of existing approaches to depression research in terms of their adoption of a hierarchy of evidence that largely stifled the application of multiple sources of knowledge, use of assessments that were uni-dimensional, the medicalization and decontextualization of complex social problems and persistent euro-centrism. In such a context, the application of a pluralistic perspective has the potential to generate socially relevant research evidence through the adoption of a ‘pluralizing’ mindset that focuses on widening one’s gaze using a ‘both/and’ heuristic, questioning the rationale for any narrowness of view, and deep curiosity around difference.

### Openings for Pluralistic Inquiry

While research on psychotherapy and related practices is increasingly shaped by a pluralistic sensibility, the transition to an explicit pluralistic research paradigm is at an early stage. As a result, there are no studies that have fully embraced pluralistic principles. Nevertheless, it is appropriate, in relation the aims of the present paper, to identify some examples of practical strategies that researchers have used to take established psychotherapy research approaches in a more pluralistic direction.

The psychotherapy literature includes many systematic reviews or metasyntheses that bring together the findings from all published studies on a topic. The majority of these reviews focuses on either qualitative or quantitative studies, with the consequence that it is not possible to compare evidence generated by different methodological approaches. Reviews by Pomerville et al. (2016), Greenhalgh et al. (2018) and Wu and Levitt (2020) demonstrate how it is possible to incorporate findings of qualitative and quantitative studies in a single review. The Pomerville et al. (2016) review takes a further pluralistic step in reporting review findings in terms of contrasting interpretative themes rather than a unified model. The potential for enhancing the social relevance of reviews through involvement of stakeholders is discussed by Abrams et al. (2021). An example of how this can be accomplished can be found in De Weger et al. (2018).

A significant development in qualitative research in recent years has been the widespread adoption of the use of multiple data analysts (e.g. an independent external auditor, research team members or research participants) as a validity criterion to support the trustworthiness of findings. The methodological assumption underpinning this procedure has been that the use of multiple analysts operates as a means of reducing misunderstandings of the data that might arise when there is only a single researcher. Within the qualitative research community, this practice has been accompanied by an interest in how power differences in understanding and data interpretation between co-analysts (e.g. members of a research team or between researchers and participants) might be handled (see, for example Levitt et al., 2021a) to ensure that final consensus judgements reflect open discussion rather than domination by more powerful voices, while still recognising legitimate differences between researchers. While such respect of epistemic justice is consistent with a pluralistic perspective, what is even more valuable is also to attend to the potential meaning and significance of differences in how co-analysts make sense of qualitative data. Nuala Frost and colleagues have shown that attention to the contrasting interpretations offered by different analysts enhances the meaningfulness of findings (Frost et al., 2010; Frost, 2016). Studies building on the work of Halling and Leifer (1991) have shown that dialogue between researchers (i.e. beyond mere consensus agreement) generates new understanding. A wide range of practical strategies for enabling research participants to engage effectively in such collaborative processes has been described by Moltu et al. (2013), Hallett et al. (2017), Matheson and Weightman (2020, 2021), Fine et al. (2021) and Soggiu et al. (2021).

A final area of emergent pluralistically informed practice concerns ways of conducting research that is not only sensitive to cultural difference but actively functions to promote the interests of members of oppressed and silenced communities. How can we create a psychotherapy research discourse that allows those who are currently silenced to be heard? Postcolonial writers, such as Spivak (2003), argue that for the ‘subaltern (i.e. the person subjected to colonial rule) to speak, and be heard, they are required to use the language and concepts of the dominant group. In counselling and psychotherapy, this means using the language and theories that have been established in the West. The use of terms, such as ‘ethnopsychotherapy’, ‘indigenous therapy’ and ‘culturally adapted therapy’, reinscribe this hegemony, by implying that Euro-American psychotherapy is the ‘unmarked category’, while others are ‘ethnic’, ‘indigenous’ or ‘adapted’ (Zerubavel, 2018). Pluralistic inquiry calls for awareness of how to ensure that research participants are not subjected to this kind of discursive erasure. Examples of how this can be accomplished include a remarkable study by Gone (2021) in which he uses his own insider knowledge as a member of an indigenous community, and his professional knowledge as a psychotherapy researcher, to allow the voice of an indigenous healer to be heard in a manner that would make sense to other therapy practitioners and researchers. A study by Mehl-Madrona (2009) used a humility-based strategy in simply asking elders in an indigenous community to tell him that they thought that Western practitioners need to know in order to be helpful to them. In a study by Waddell et al. (2021), a research partnership was built up through joint participation in indigenous spiritual rituals over an extended period, prior to data collection and analysis.

The examples of openings for pluralistic inquiry outlined in this section are not intended as a comprehensive account of how to conduct pluralistic research or reviews on psychotherapy topics. Rather, the intention has been to show how a pluralistic perspective builds on existing methodologies in ways that allow difference to become a focus of interest.

## A Pluralistic Perspective on Evidence

Within the domain of psychotherapy policy, research and practice, one of the most significant implications of adopting a pluralistic perspective is that it invites further consideration of what counts as evidence. There is broad agreement within professional communities, and society as a whole, around the value of evidence-based practice (EBP). The most widely cited definition of EBP within the field of medicine describes EBP in terms that are consistent with a pluralistic standpoint that acknowledges multiple perspectives and stakeholders:

…the conscientious, explicit and judicious use of current best evidence in making decisions about the care of the individual patient. It means integrating individual clinical expertise with the best available external clinical evidence from systematic research [and]… thoughtful identification and compassionate use of individual patients’ predicaments, rights, and preferences (Sackett et al., 1996, p. 71).

A similarly pluralistic stance in relation to evidence is apparent in an APA policy position that resulted from years of debate within the field of psychotherapy research:

Evidence-based practice in psychology (EBPP) is the integration of the best available research with clinical expertise in the context of patient characteristics, culture, and preferences (American Psychological Association, 2006, p. 273)

When practising in evidence-based way, the APA recommends that the practitioner should draw on a wide range of knowledge sources that may be relevant for each particular case, including:

…clinical observation, qualitative research, systematic case studies, single-case experimental designs, public health and ethnographic research, process-outcome studies, studies of interventions as these are delivered in naturalistic settings (effectiveness research), RCTs and their logical equivalents (efficacy research), and meta-analysis (Levant, 2005, pp. 7–8).

The underlying principles of EBPP are radically different from defining evidence in terms of specific methods (e.g. experimental designs, such as RCTs). Instead, the statements cited above advise that clinical decisions should be made in a collaborative manner that takes account of the local context and cultural beliefs and preferences of each client and that information about research findings associated with different methodologies being made equally available to clinicians.

A pluralistic perspective goes further than these EBPP principles, by opening up an appreciation not only of collaborative decision-making around the application of research evidence but also collaborative co-production and critique of the evidence itself. In large part, the medical research being considered by Sackett et al. (1996) largely consisted of findings from laboratory science and drugs trials that could only be fully understood by a relatively small number of specialist researchers. By contrast, most research studies of psychotherapy are intelligible, and of interest, to a wide potential readership of therapists, clients and other stakeholders. In a study by McPherson et al. (2020) mentioned earlier, when the design, procedures and results of a psychotherapy RCT were explained to service users and other stakeholders, they were highly sceptical of the value of evidence that it provided. The issue of the credibility of evidence from psychotherapy research can be understood as arising from the fact that any research study generates a set of truth claims that are grounded and warranted in terms of methodological procedures that have been followed (Toulmin, 1958). For example, clients have been helped by therapy because they exhibited reduction in depression scores as measured by the BDI which had been administered and analysed in a competent manner by trained researchers. However, therapists or clients who read such a study may have other grounds and warrants available to them, such as an understanding of depression that is different from BDI item content, or other explanations for why scores changed over time (McLeod, 2021). When non-researchers closely scrutinise psychotherapy research studies (as in McPherson et al., 2020) it becomes apparent that the truth claims that they encounter in such research reports are to a large extent only warrantable within the narrow parameters of specific research and practice sub-cultures and readily fall apart when exposed to truth claims arising from lived experience.

A pluralistic perspective on psychotherapy research suggests that the quality and credibility of evidence that is available to inform policy and practice would be enhanced by wider dialogue around the design and conduct of studies, data analysis and the meaning and implications of research findings. This dialogue can take place between groups of researchers and across academic disciplines and also between the research community and any other people and groups (clients, practitioners, members of the public and interest groups) who have a stake in making sure that the therapy that is being offered is relevant to the needs of individuals and communities. The relative absence of such dialogue, at the present time, can be understood as representing a form of epistemic injustice that has been described as a manifestation of ‘strategic ignorance’: the process through which members of privileged groups in society retains epistemic control by ‘knowing what not to know’ (McGoey, 2010, 2019).

In the wider field of healthcare, the limitations of defining evidence in terms of specific methods, such as RCTs and systematic meta-analysis reviews, have been recognised as contributing to difficulties in providing individualised patient-centred care. An important strand of this evolving critique has been the analysis of the implications of basing research in a narrow conceptualisation of causality, alongside a growing awareness of the possibilities associated with a pluralistic understanding of causes that draws on concepts, such as affordances, dispositions and vectors (Anjum et al., 2020). A flexible, conceptually rich framework for making sense of everyday causality already exists within behavioural psychology (Haynes et al., 2012). From a pluralistic perspective, as well as embracing methodological diversity and stakeholder dialogue, the task of enhancing the relevance and sensitivity of research evidence needs to consider the implications of different ideas about causality for the conduct and analysis for all research designs.

Demonstrating the practical societal relevance of co-produced forms of evidence represents a major challenge for those who support the adoption of a more pluralistic approach to psychotherapy research. Currently, we are in a situation in psychotherapy research in which the training received by most researchers limits their understanding of the wide diversity of research approaches that exist. In addition, grant agency boards are filled with researchers who have established their reputations on the basis of expert implementation of a similarly restricted set of established methodologies, and the procedures of grant agencies and governmental guideline commissioning groups generally use an evidence hierarchy framework to inform their decision-making. The views of such sector leaders are unlikely to be swayed by academic debate around research methodology and values. To make an impact on business as usual within psychotherapy research policy and practice, it is necessary to produce actual research findings that demonstrably make a difference.

As discussed earlier, one of the guiding principles of pluralistically informed inquiry is the intention to carry out research that is oriented towards the accomplishment of social justice goals. What this means is that, from a pluralistic perspective, research evidence is evaluated in terms of the extent to which it contributes to creating a better society, alongside whatever technical validity criteria and theory-building aims that may be applicable. Methods for evaluating the extent to which programmes of research accomplish social goals are not well-developed. Nevertheless, at the present time, it is hard to argue that more than 70years of psychotherapy research have led to an improvement in the effectiveness of psychotherapy or the reduction of mental health problems in society. Analysis of historical trends in psychotherapy outcomes has not shown that therapy has become more effective, even in areas of practice that have been supported by considerable investment in research, such as CBT for depression (Johnsen and Friborg, 2015), psychotherapy for problems reported by young people (Weisz et al., 2019) and suicide prevention (Fox et al., 2020). Leading figures in psychotherapy research have argued that the difficulties in applying RCTs in psychotherapy contexts mean that evidence generated by this methodology is best by a wide range of potential biases that are hard to control (Cuijpers et al., 2019a,b, 2020). It has also been suggested that RCT evidence lacks relevance for the development of the kind of service provision that is likely to appropriate to future social needs (van Os et al., 2019). In addition, among those RCTs that have been most influential in setting the agenda for therapy policy and provision, few have ever been replicated, and most stand out as outliers in terms of reporting more positive findings than other similar studies (Frost et al., 2020).

A pluralistic perspective makes it possible to re-vision the types of evidence that can be used to inform psychotherapy practice. For example the logic of a collaborative style of research is consistent with initiatives that use research tools and strategies to enable specific psychotherapy provider organisations, or networks of clinics in a particular city or region, to collect and analyse data from clients and other stakeholders in the context of on-going action research that aims to generate enhanced mental health outcomes at a community level. At the present time, the assumption that service improvement requires the top-down application of generalised knowledge from RCTs has meant that such ground-up projects have rarely been attempted on a sustained basis. Within a 3–5year period, a pluralistically informed action MMR study along these lines, that involved co-productive research alliances with clients, practitioners and community groups, might be able to demonstrate tangible effects on social wellbeing and cultural capital that would be hard to for funders and policy-makers to ignore.

In terms of the type of evidence that is produced by collaborative and co-produced studies, a significant consequence of greater involvement of clients, practitioners and other stakeholders will be that research findings will become more contextualised. On the whole, the type of knowledge that academic researchers bring to the inquiry process is more focused on theoretical perspectives, whereas the knowledge and interest of community-based stakeholders are more focused on the specific local context with which they are familiar. Greenhalgh and Manzano (2021) discuss the ways in which attention to context can enhance the practical relevance of research.

## Conclusion

The aim of this paper has been to highlight some of the ways in which a pluralistic philosophical stance might enhance the practical value and social relevance of research in psychotherapy and related disciplines. We continue to be surprised by what is uncovered by a pluralizing way of thinking. We are continually challenged by the interpersonal skills and social courage that entailed in a pluralistic perspective, and encourage readers to view our ideas as a starting point and invitation to collaboration and further dialogue. Our experience has been that a pluralistic perspective has heighted our appreciation of the value of existing methodologies. Just as pluralistic therapy provides a framework for channelling existing therapeutic ideas and methods in the service of helping a client to attain their life goals, a pluralistic perspective on research similarly regards existing methodologies as invaluable resources to be cherished and used as appropriate. The therapy research community has created a massive array of research tools (see, for example Liamputtong, 2019). A pluralistic perspective does not seek to re-invent these techniques but merely to offer some ideas about how they can be most effectively combined and deployed.

Pluralism offers a philosophical and conceptual meta-model that can be used as a guide (along with other meta-perspectives) to thinking about long-term research objectives and purposes. Pluralism also opens up a wide range of concrete activities, projects and practices that can be pursued immediately. Examples of achievable, low-cost pluralistically oriented research initiatives include as: experimenting with open review/comment journal publishing; conducting pluralistically informed research reviews that incorporate evidence not only from different methodologies but also make use of review teams with different cultural backgrounds and life experience; activating co-production at a local level through collaborative projects that use research to enhance practice in specific agencies/clinics; and learning with and from other disciplines, occupational groups and community organisations through joint seminars and workshops that share experience in co-production, decolonising, and strategies for working constructively with difference.

To move away from euro-centrism, and profession-centrism, the psychotherapy research community needs to do more to recognise forms of practice beyond existing professional labels. There are many places in the world where psychotherapy is not professionalised but where people nevertheless help others through various psycho-social interventions, practices and rituals (Zacharias, 2006). A pluralistic perspective aims to include these practices, perspectives, concepts and principles and recognises their value and potential enrichment of both theory and practice. At the same time, pluralism also takes a critical stance by reflecting on whether these practices and ideas should be subsumed within its own discourse. It can also highlight potentially problematic appropriations. For example some therapeutic schools have borrowed concepts and methods from other cultures (e.g. mindfulness and Morita therapy), but often stripped them of the cultural context, omitting the voices of the people who offered them and developed the ideas around them. Pluralistic inquiry can offer an antithesis to research that whitewashes concepts and methods borrowed from other cultures by revealing their cultural embeddedness. Western concepts and practices of psychotherapy are often implanted without adapting them to cultural contexts, effectively marginalising local knowledge of healing (Sidhu, 2017). Through a pluralistic perspective, researchers can develop dialogues and use tools that support practitioners to develop counselling practices on the basis of indigenous cultural strengths and resources.

Finally, we suggest that it is essential to highlight the potential broader outcomes of pluralistically oriented research, beyond the specific domain of therapy theory and practice. All peoples and cultures are bound together in a collective need to change our way of life and relationship with nature in order to create ways of surviving the inevitable climate crisis that we have brought about. Core elements of that dysfunctional way of life include racism, colonialism, slavery/trafficking and militarism. Erosion of democratic processes represents a key element in the on-going failure to address these crises. Adoption of a pluralistic approach to therapy research has the potential to help us, as psychotherapists, mental health practitioners and researchers, to develop ways of understanding and conducting collective inquiry that provide all participants with awareness and skills around shared decision-making, listening to others, working together, live with complexity and uncertainty and be willing to stand up for collective values and justice. Along with re-visioned therapy practices, these research outcomes represent some of the ways in which we might hope to be able to support individuals, families and communities to engage in active citizenship.

## Data Availability Statement

The original contributions presented in the study are included in the article/supplementary material, and further inquiries can be directed to the corresponding author.

## Author Contributions

All authors listed have made a substantial, direct and intellectual contribution to the work, and approved it for publication.

## Conflict of Interest

The authors declare that the research was conducted in the absence of any commercial or financial relationships that could be construed as a potential conflict of interest.

## Publisher’s Note

All claims expressed in this article are solely those of the authors and do not necessarily represent those of their affiliated organizations, or those of the publisher, the editors and the reviewers. Any product that may be evaluated in this article, or claim that may be made by its manufacturer, is not guaranteed or endorsed by the publisher.

## Footnotes

^1^ www.nice.org.uk
